# A Comprehensive Review of Nutritional Influences on the Serotonergic System

**DOI:** 10.1016/j.advnut.2025.100524

**Published:** 2025-09-23

**Authors:** Justin Tang, Luke Krushelnycky, Abir Shaqo, Clara E Cho

**Affiliations:** 1 Department of Biomedical Sciences, University of Guelph, Guelph, ON, Canada; 2 Department of Human Health Sciences, University of Guelph, Guelph, ON, Canada

**Keywords:** serotonin, 5-HT, nutrition, diet, macronutrients, micronutrients, gut microbiota, health, pathologies, therapeutics

## Abstract

Serotonin (5-hydroxytryptamine) is a critical monoamine neurotransmitter and hormone that orchestrates a vast array of physiological and psychological processes, including mood, sleep, appetite, and gastrointestinal motility. Serotonin synthesis is dependent on the availability of its dietary precursor, the essential amino acid tryptophan, and it affects biochemical pathways that may be modulated by other nutrients. We conducted a literature search to comprehensively examine the nutritional influences on the serotonergic system. Relevant original research, systematic reviews, meta-analyses, and clinical trial reports were retrieved from PubMed, Scopus, and Google Scholar, with additional articles identified from reference lists of published review papers. Key nutritional determinants of serotonergic function include macronutrients that influence the tryptophan-to-large neutral amino acid ratio (a regulator of tryptophan availability in the brain) and micronutrients, such as B-vitamins, vitamin D, iron, and magnesium, that serve as essential cofactors in serotonin synthesis and metabolism. Emerging evidence also highlights the role of the gut microbiota, shaped by dietary components, prebiotics, and probiotics, in modulating serotonergic function across both central and peripheral systems. Nutritional factors that affect serotonin have been increasingly linked to conditions such as depression, anxiety, sleep disturbances, disordered eating, obesity, and irritable bowel syndrome. Altogether, this review emphasizes the profound impact of nutrition on serotonergic regulation and advocates for targeted dietary approaches as promising catalysts for optimizing human health. Key research gaps and future directions are outlined to help advance the translation of current evidence into precise nutritional guidelines and clinical applications, with the complexity of serotonin pathways as an important consideration.

**Statement of Significance**

Serotonin affects many physiologic processes in the body, yet studies investigating the impact of nutrition in the regulation of serotonin-related outcomes have not been integrated. This review synthesizes emerging evidence on both macronutrient and micronutrient influences on serotonin pathways, connecting central and peripheral mechanisms including gut–brain interactions, presenting a new perspective on nutritional targets and translational potential for various serotonin-related diseases, spanning mental, metabolic, and gastrointestinal outcomes.

## Introduction

Serotonin, or 5-hydroxytryptamine (5-HT), is a phylogenetically conserved signaling molecule with diverse roles in virtually all living organisms [1]. In mammals, serotonin functions as a neurotransmitter within the central nervous system (CNS), a local hormone in the gut, a vasoactive agent, and a growth factor [2,3]. Within the CNS, serotonergic neurons originate primarily from the raphe nuclei and send projections throughout the brain, where they modulate critical functions, including mood, anxiety, sleep–wake cycles, appetite, cognition, learning, and memory [4]. Peripherally, the vast majority (∼90%–95%) of the body’s serotonin is synthesized and stored within enterochromaffin (EC) cells lining the gastrointestinal tract. Gut-derived serotonin can act as a key regulator of gut motility, secretion of intestinal fluids and mucus, sensation, and inflammation [5,6]. Given its pervasive influence across both central and peripheral systems, dysregulation of the serotonergic system is implicated in the pathophysiology of a wide spectrum of neuropsychiatric and peripheral disorders. These include major depressive disorder (MDD) [7], anxiety disorders [8], sleep disturbances [9], and metabolic diseases [10,11], as well as irritable bowel syndrome (IBS) [12].

A unique feature of serotonin biosynthesis is its dependence on the availability of its precursor, the essential amino acid l-Trp, which must be obtained exclusively from dietary sources [13]. This inherent link establishes a direct biochemical pathway through which nutrition can exert profound effects on serotonin concentrations and subsequent function. However, the influence of nutrition extends beyond Trp availability alone. The broader nutritional landscape, encompassing macronutrient balance, micronutrient adequacy, and even the composition and metabolic activity of the gut microbiota, plays a complex and multifaceted role in regulating serotonin homeostasis [14,15].

Identifying the nutritional modulators of serotonin is of paramount importance for several reasons. It not only provides key insights into the pathophysiology of serotonin-related disorders but also illuminates potential avenues for developing novel, nonpharmacological, and preventative strategies centered on nutritional interventions. Although pharmacological agents targeting the serotonin system, such as selective serotonin reuptake inhibitors (SSRIs), represent cornerstone treatments for many conditions, they are not universally effective and can be associated with undesirable side effects [16]. Nutritional approaches, whether employed as adjuncts to conventional therapies or as standalone interventions, could offer a more holistic, personalized, and potentially safer means of supporting serotonergic health and overall well-being.

The purpose of this review is to comprehensively examine the nutritional influences on the serotonergic system across central and peripheral compartments. We will first introduce the biochemical pathways of serotonin synthesis, metabolism, transport, and signaling. Subsequently, we will discuss key nutritional modulators of serotonin, bringing attention to macronutrients, micronutrients, phytochemicals, and those that alter the gut microbiota. Finally, we will explore the clinical implications for human health and disease and highlight key research gaps and future directions for optimizing serotonin function through nutrition.

## Methods

A literature search was performed to gather relevant peer-reviewed published scientific articles for this narrative review. Major scientific databases, including PubMed, Scopus, and Google Scholar, were searched from their inception to May 2025. The search strategy employed a combination of keywords and subject headings related to the core topics. Key search terms included but were not limited to “serotonin,” “5-hydroxytryptamine,” “5-HT,” “tryptophan,” “nutrition,” “diet,” “macronutrients,” “carbohydrates,” “protein,” “fats,” “micronutrients,” “B-vitamins,” “vitamin D," “iron,” “magnesium,” “zinc,” “gut microbiota,” “probiotics,” “prebiotics,” “phytochemicals,” “fermented foods,” “depression,” “anxiety,” “sleep disorders,” “obesity,” and “irritable bowel syndrome.”

The selection of articles included original research (both human and animal studies) not restricted by study design, systematic reviews, meta-analyses, and clinical trial reports. Although there was no strict date cutoff, priority was given to publications from the last 15 y to ensure the inclusion of current evidence. The reference lists of key review articles were also manually screened to identify additional relevant publications. The final selection of literature was based on its direct relevance to the topics of this review, covering the biochemistry, nutritional modulation, and clinical implications of the serotonergic system.

## Serotonin Biochemistry: Synthesis, Degradation, Transport, and Signaling

Serotonin metabolism involves distinct synthesis, degradation, transport, and signaling processes, with modifications that can be afforded by nutritional factors. This section discusses the biochemical pathways governing serotonin and key enzymes for the purpose of building context for manifold impacts of nutrition.

### Serotonin synthesis pathway

**FIGURE 1:**
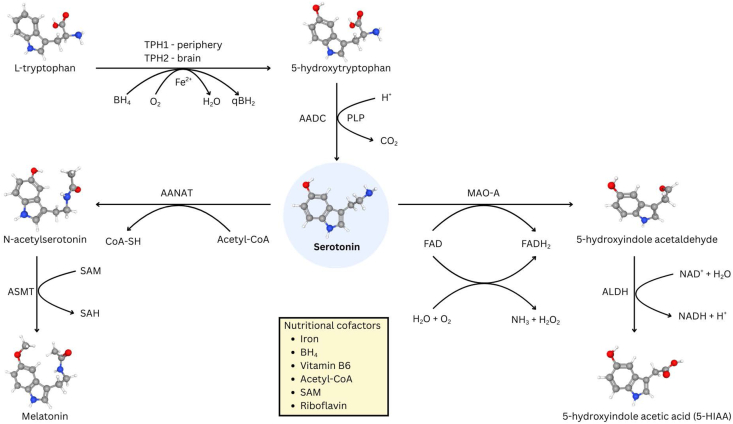
The biosynthesis of serotonin and metabolic products.l-Tryptophan is converted to 5-hydroxytryptophan (5-HTP) by tryptophan hydroxylase [tryptophan hydroxylase (TPH) 1 in periphery, and TPH2 in brain)] in a reaction requiring molecular oxygen (O_2_), Fe^2+^, and tetrahydrobiopterin (BH_4_). 5-HTP is then decarboxylated to serotonin by aromatic L-amino acid decarboxylase (AADC), which requires pyridoxal-5′-phosphate (PLP, active vitamin B6). Serotonin can be *N*-acetylated by arylalkylamine *N*-acetyltransferase (AANAT) in an acetyl-CoA-dependent step and subsequently *O*-methylated by acetylserotonin *O*-methyltransferase (ASMT) to form melatonin in the pineal gland using *S*-adenosyl-l-methionine (SAM), which subsequently is converted to *S*-adenosylhomocysteine (SAH). Alternatively, serotonin can undergo oxidative deamination predominantly by monoamine oxidase A (MAO-A), which contains a covalently bound flavin adenine dinucleotide (FAD), to produce 5-hydroxyindole acetaldehyde followed by oxidation by aldehyde dehydrogenase (ALDH) to yield 5-hydroxyindoleacetic acid (5-HIAA). Nutritional cofactors (iron, BH_4_, vitamin B6, acetyl-coenzyme A (actyl-CoA), SAM, and riboflavin-derived FAD) are therefore critical at multiple steps. qBH_2_, quinonoid dihydrobiopterin; SH, thiol group.

Serotonin is synthesized from the essential amino acid Trp in a tightly regulated 2-step enzymatic process (Figure 1). The initial and rate-limiting step involves the hydroxylation of Trp at the 5-position of the indole ring to form 5-hydroxytryptophan (5-HTP). This reaction is catalyzed by the enzyme Trp hydroxylase (TPH) [17]. TPH activity is dependent on several cosubstrates and cofactors, including molecular oxygen, ferrous iron (Fe^2+^), and tetrahydrobiopterin (BH_4_) [18]. Notably, 2 distinct isoforms of TPH exist with tissue-specific expression patterns: TPH1 is predominantly expressed in peripheral tissues, including the gut EC cells and the pineal gland, whereas TPH2 is primarily localized within the serotonergic neurons of the brainstem raphe nuclei in the CNS [19,20]. This compartmentalization allows for differential regulation of the central and peripheral serotonin pools. After its formation, 5-HTP is decarboxylated to yield serotonin by the enzyme aromatic l-amino acid decarboxylase (AADC) [21], which is a relatively promiscuous enzyme also present in catecholaminergic neurons for conversion of l-3,4-dihydroxyphenylalanine into dopamine. AADC requires pyridoxal-5′-phosphate (PLP), the active form of vitamin B6, as an essential cofactor for its catalytic activity [22].

### Serotonin degradation

The primary catabolic pathway for serotonin involves oxidative deamination, which is catalyzed by monoamine oxidase A (MAO-A), to form the intermediate 5-hydroxyindoleacetaldehyde. This unstable aldehyde is subsequently oxidized by aldehyde dehydrogenase to the major inactive metabolite, 5-hydroxyindoleacetic acid (5-HIAA), which is readily excreted in the urine [23]. Importantly, MAO-A utilizes FAD, derived from riboflavin (vitamin B2), as a cofactor, highlighting another potential point of nutritional influence [24]. In the pineal gland, serotonin *N*-acetyltransferase can convert serotonin to *N*-acetylserotonin, from which hydroxyindole *O*-methyltransferase, using *S*-adenosyl-l-methionine, generates melatonin, a hormone produced principally at night [25,26]. Both *N*-acetylserotonin and melatonin display circadian rhythms, surging after the onset of darkness and decreasing before the onset of light in the morning [27]. In addition to being a precursor, serotonin is suggested to act as an autocrine signal in the pineal gland, sensitizing melatonin release with fine modulation by other neurotransmitters, including norepinephrine [28].

### into the brain

**FIGURE 2:**
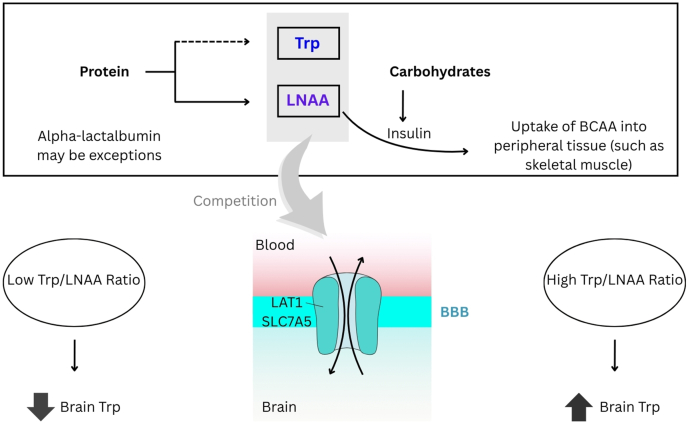
Regulation of l-tryptophan (Trp) transport across the blood-brain barrier (BBB) by protein vs. carbohydrates. A high-protein meal provides Trp along with an even greater abundance of large neutral amino acids (LNAAs). Trp and LNAAs are in competition for transport across the BBB via the L-type amino acid transporter 1 (LAT1), also known as solute carrier family 7 member 5 (SLC7A5). The net result is a reduction in the plasma Trp/LNAA ratio and greater brain Trp uptake for serotonin synthesis. However, specific protein sources such as α-lactalbumin may represent exceptions, as specific protein hydrolysates can be rich in Trp relative to other LNAAs, which may increase the Trp/LNAA ratio. A high-carbohydrate meal stimulates insulin release, which promotes the uptake of branched-chain amino acids (BCAAs, a subset of LNAAs) into skeletal muscle. This reduces plasma LNAA concentrations, thereby increasing the Trp/LNAA ratio and enhancing brain Trp uptake, making more precursor available for serotonin synthesis.

For serotonin synthesis to occur within the CNS, its precursor, Trp, must first traverse the blood-brain barrier (BBB). This transport is mediated by the L-type amino acid transporter 1 (LAT1), also known as solute carrier family 7 member 5 (SLC7A5), which functions as a sodium-independent exchanger protein [29]. Trp competes with other large neutral amino acids (LNAAs), namely, Tyr, Phe, Val, Leu, and Ile, for LAT1 binding sites [30]. Consequently, brain Trp uptake, and thus CNS serotonin synthesis, is determined by the plasma ratio of Trp to the sum of these competing LNAAs (Trp/LNAA ratio) rather than by absolute plasma Trp concentrations [31] (Figure 2). Insulin stimulated by carbohydrate intake can also indirectly enhance Trp transport by reducing competing LNAAs in circulation [32]. Furthermore, the activity of LAT1 (SLC7A5) can be influenced by cellular energy status and membrane lipid composition [33,34]. Thus, regulatory mechanisms for LAT1 at the BBB are complex and include expression levels of the transporter, and little is known about direct nutrient-mediated regulation of transporter activity itself beyond substrate competition [35].

### Serotonin receptors and signaling

Once synthesized and released, serotonin exerts its diverse effects by binding to a large family of specific receptors. At least 14 distinct serotonin receptor subtypes have been identified in mammals, grouped into 7 families (5-HT1 to 5-HT7 receptors) based on their structural, pharmacological, and signaling properties [36]. Most of these are G protein-coupled receptors, with the exception of the 5-HT3 receptor, which is a ligand-gated ion channel [37]. For example, 5-HT1A receptors, often acting as autoreceptors on serotonergic neurons or as postsynaptic receptors in regions such as the hippocampus and amygdala, are typically coupled to Gi/o proteins, leading to inhibition of adenylyl cyclase and modulation of ion channels [38]. Conversely, 5-HT2A receptors, widely expressed in the cortex, are coupled to Gq/11 proteins, activating phospholipase C and leading to intracellular calcium mobilization [39]. Other important subtypes include 5-HT1B/1D, 5-HT2B, 5-HT2C, 5-HT3, 5-HT4, 5-HT6, and 5-HT7 receptors, each with distinct signaling cascades and physiological roles such as vasoconstriction, mood, appetite, nausea, gut motility, cognition, and circadian rhythms [40]. The specific distribution and signaling pathways of these receptor subtypes underpin serotonin’s multifaceted influence on brain function and peripheral physiology and can be indirectly influenced by nutritional factors that alter serotonin availability or neuronal membrane environments.

## Macronutrient Influences on Serotonin

The macronutrient composition of the diet, specifically the relative amounts of protein, carbohydrates, and fats, exerts significant, albeit sometimes indirect, influence over serotonin availability and synthesis, primarily through modulation of Trp transport and precursor supply.

### Protein

Protein serves as the sole dietary source of the essential amino acid Trp. Paradoxically, however, the consumption of a high-protein meal typically leads to a decrease, rather than an increase, in brain Trp uptake and serotonin synthesis [41]. This counterintuitive effect arises because dietary protein, although supplying Trp, provides an even greater abundance of the other LNAAs (Tyr, Phe, Val, Leu, and Ile) that compete with Trp for transport across the BBB via the LAT1 transporter [42]. The net result of ingesting a typical protein-rich food is therefore a reduction in the plasma Trp/LNAA ratio, which limits Trp entry into the brain and consequently curtails CNS serotonin production. Nevertheless, specific protein sources or specially formulated Trp-enriched protein hydrolysates might represent exceptions. For example, α-lactalbumin, a whey protein fraction notably rich in Trp relative to other LNAAs, has been shown in some studies to potentially increase the Trp/LNAA ratio and enhance markers of serotonin function [43,44]. Thus, the source and amino acid profile of dietary protein can be critical determinants of its ultimate impact on brain serotonin precursor availability.

### Carbohydrates

Carbohydrates represent perhaps the most well-established dietary factor influencing brain Trp uptake and subsequent serotonin synthesis [45]. The ingestion of carbohydrates triggers the secretion of insulin from pancreatic β-cells. Although the primary role of insulin is to facilitate glucose uptake into peripheral tissues, it also potently stimulates the uptake of most LNAAs, particularly branched-chain amino acids (Val, Leu, and Ile), into skeletal muscle [46]. Trp, however, is unique among LNAAs as a significant portion circulates bound to albumin, making its peripheral uptake less sensitive to insulin’s direct action [47]. Consequently, after a carbohydrate-rich, relatively protein-poor meal, the plasma concentrations of competing LNAAs decrease more substantially than plasma Trp concentrations. This differential effect leads to an increase in the Trp/LNAA ratio, thereby facilitating Trp transport across the BBB and enhancing the substrate pool available for serotonin synthesis within the CNS [31,48] (Figure 2). This biochemical mechanism is frequently invoked to explain the purported “comfort food” effect associated with carbohydrate-rich meals, potentially mediated by a transient increase in brain serotonin concentrations that may positively influence mood [49]. However, it is important to consider that the magnitude of this effect can be influenced by other variables. For example, the type of carbohydrate consumed (e.g., simple compared with complex) determines the speed and peak of the insulin response, with simple sugars inducing a more rapid but transient effect [50]. The timing of preload and the composition of the entire meal are also critical; consuming protein alongside or before carbohydrates can introduce competing LNAAs, thereby blunting the increase in the Trp/LNAA ratio [50].

Conversely, in physiological states characterized by low insulin concentrations, such as patients with type 1 diabetes, the beneficial effect of carbohydrates on the Trp/LNAA ratio is lost [51]. Low insulin fails to promote the peripheral uptake of competing LNAAs, which can lead to a reduced Trp/LNAA ratio and, consequently, diminished Trp transport into the brain. This highlights the role of insulin in determining central serotonin precursor availability and suggests that conditions of poor metabolic control could negatively impact serotonergic function [51].

### Fats

The direct role of dietary fats in modulation of serotonin synthesis is less clearly defined compared with that of protein and carbohydrates. However, there is growing recognition of the role of the lipid environment in the function and location of membrane-bound proteins, including those specific to serotonin, as the interaction between lipids and proteins may contribute to serotonergic synaptic efficacy [52]. Long-term dietary fat intake patterns, particularly concerning the type of fatty acids consumed, can differentially influence serotonin receptor and transporter binding in the rat brain [53]. ω-3 (n–3) PUFAs, notably EPA and DHA, which are enriched in fatty fish, are integral structural components of neuronal cell membranes [54]. Several lines of evidence suggest that ω-3 PUFAs may enhance serotonergic neurotransmission through various mechanisms. These include increasing cell membrane fluidity, which may shift the equilibrium toward an active form of the receptor, making serotonin binding more favorable [55]; modulating the expression of serotonin receptors [56]; and influencing the production of eicosanoids and other lipid mediators via neuroinflammatory pathways, which can affect serotonin release [56,57]. Serotonin itself can also induce alterations in the lipid composition, which is thought to further affect serotonergic signaling as a receptor-independent pathway for serotonin action [58].

## Micronutrient Influences on Serotonin

Beyond macronutrients, several micronutrients (vitamins and minerals) play indispensable roles as cofactors or modulators within the serotonin synthesis and metabolic pathways (Figure 1). Consequently, deficiencies in these micronutrients can directly impair serotonin production or alter its turnover, potentially contributing to clinical manifestations associated with serotonergic dysfunction.

### B-Vitamins

Several B-complex vitamins play key roles in serotonin metabolism. The active form of vitamin B6, PLP, serves as an essential cofactor for AADC, the enzyme responsible for the final conversion of 5-HTP to serotonin [22]. In preclinical models, an accumulation of the intermediate 5-HTP and lower serotonin concentrations have been reported with semicarbazide-induced PLP deficiency in rats establishing ∼50%–60% PLP concentration of control [59], whereas administration of 10 mg/kg pyridoxine hydrochloride has been shown to enhance the rate constant for utilization of radiolabeled 5-HTP for serotonin formation in the monkey brain [60]. Disturbances in pineal function because of decreased synthesis of melatonin (attributed to lower serotonin concentrations) have also been reported in rat studies, suggesting a possible connection between vitamin B6 deficiency and alterations in sleep pattern and mood [61]. In humans, vitamin B6 status is typically determined by measuring PLP concentrations, with values <20 nmol/L indicating deficiency and 20–30 nmol/L indicating suboptimal status [62], although it is important to recognize that various factors unrelated to vitamin B6 status can affect plasma PLP [63]. To date, human studies that directly assess serotonin synthesis in the context of vitamin B6 status are lacking. However, individuals with PLP concentrations <30 nmol/L have been shown to exhibit more frequent symptoms of depression, which has been theoretically linked to the role of PLP in the Trp–serotonin pathway [64]. Extending further, neuroactive and immunomodulatory compounds are also known to be produced in PLP-dependent reactions of the kynurenine pathway, highlighting vitamin B6 as a plausible metabolic checkpoint in regulating Trp flux and influencing downstream processes with potential clinical applications [65].

Vitamin B9 (folate) and vitamin B12 (cobalamin) have been raised as possible candidates for neuromodulation through their effects on serotonin biosynthesis, neuronal activity, or associated metabolic pathways including one-carbon metabolism in the generation of the universal methyl donor, *S*-adenosylmethionine (SAM) [[66], [67], [68], [69]]. As previously noted, BH_4_ is an indispensable coenzyme for TPH, the rate-limiting enzyme in the serotonin synthesis cascade. Folate in the form of 5-methyl-tetrahydrofolate (5-methyl-THF) is involved in regenerating BH_4_, which is highly susceptible to oxidation [66]. A recent study using *Caenorhabditis elegans* has also revealed a metabolism-independent role for folate receptor-1 in the activation of serotonergic neurons to regulate behavior in response to 10-formyl-THF, but not 5-methyl-THF, 5-formyl-THF, and THF, indicating the specificity arising from folate forms [67]. Further, the stimulatory effect of SAM has been proposed to be central to the concentration of serotonin [68], as inadequate SAM synthesis from vitamin B12 deficiency has been suggested to yield lower serotonin synthesis [69].

Tryptophan also serves as a precursor for de novo synthesis of vitamin B3 (niacin) via the kynurenine pathway [70]. In situations of inadequate dietary niacin intake, a larger proportion of dietary Trp may be diverted toward niacin production to meet the body’s needs and may reduce the amount of Trp available for serotonin synthesis. However, this competition is generally considered clinically relevant only in cases of severe niacin deficiency, such as pellagra [70]. Finally, vitamin B2 (riboflavin), as a component of FAD, acts as a cofactor for MAO-A, the primary enzyme responsible for serotonin degradation [24]. Although a deficiency could theoretically slow serotonin breakdown, potentially leading to higher concentrations at the synapse, the direct clinical relevance of riboflavin status specifically to serotonin-related mood disorders, as distinct from its role in general cellular energy metabolism, is less clearly established.

### Vitamin D

A growing body of evidence supports the role of vitamin D in various aspects of brain function, including the regulation of serotonergic pathways. Vitamin D receptor and 1α-hydroxylase, the enzyme responsible for synthesizing the active form of vitamin D, 1,25-dihydroxyvitamin D3 (1,25(OH_2_)D_3_), are widely distributed across the prefrontal cortex, cingulate gyrus, hippocampus, and hypothalamus, which are areas of the brain implicated in cognitive processes, emotion, and behavior [71]. Preclinical studies using cell cultures and animal models have demonstrated that 1,25(OH_2_)D_3_ results in higher serotonin concentrations, consistent with the upregulation of *Tph2* gene expression via the vitamin D response element located within the promoter region of the *Tph2* gene [72], in concert with the repression of serotonin transporter (SERT) and MAO-A [73].

Vitamin D insufficiency is generally defined as a serum 25-hydroxyvitamin D concentration of 21–29 ng/mL, whereas concentrations <20 ng/mL constitute frank deficiency [74]. As vitamin D insufficiency and low central serotonin concentrations have been suggested to form a common denominator in elevated neuropsychiatric disease risk [75], a link supported by large-scale epidemiological studies associating low serum 25-hydroxyvitamin D concentrations with a higher incidence of depression [76], the ability of 1,25(OH_2_)D_3_ to amplify serotonergic actions may represent a therapeutic candidate for steering neurological control [76]. In peripheral tissue, the repressive effect of vitamin D on TPH1 has previously been found consistent with lower serotonin production in the gut [77] and has been implicated with the regulation of melatonin concentrations, adding further dimensions to sleep and mood indicators in relation to the serotonergic system [78].

### Minerals

Inorganic minerals (iron, magnesium, and zinc) serve as cofactors for a wide range of biochemical reactions and are modulators of serotonin metabolism. Iron, in its ferrous (Fe^2+^) state, functions as a cofactor for TPH, the rate-limiting enzyme in serotonin synthesis [79]. Iron deficiency, recognized as one of the most prevalent micronutrient deficiencies globally [80], is characterized by various clinical symptoms including anemia, fatigue, growth retardation, disturbed immune function, and cognitive impairment [81]. The link between iron and neuronal outcomes is particularly relevant in the context of iron deficiency, which may partly be mediated by the reduction in serotonin and other iron-dependent neurotransmitter synthesis pathways [81]. In line with this, rats fed a diet with low iron content (18–20 mg/kg) had reduced serotonin concentrations, which may be attributed to the downregulation of the iron-dependent enzyme TPH because brain iron content and alterations in serotonin metabolism did not recover despite iron supplementation (390 mg/kg) [82]. However, others have also reported elevated serotonin concentrations [83], as well as lower densities of SERT in the striatum [84] and reduced serotonin uptake [85], all of which may indicate the role of iron in modulating the availability of neurotransmitter in different compartments of metabolism.

Magnesium has a fundamental role in the regulation of neuronal transmission [86] as well as many aspects of neurobiological functions [87], in addition to being essential for major metabolic reactions, particularly those requiring ATP [88]. Magnesium deficiency has been associated with increased anxiety and depressive symptoms in both animal models and human studies, and magnesium supplementation has demonstrated potential antidepressant effects in some clinical trials [89,90]. The importance of an intact serotonergic system has been shown by the elimination of the antidepressant action of magnesium in animals pretreated with an inhibitor of serotonin synthesis [91]. Interactions among several systems may also serve as a potential mechanism by which magnesium alters neurologic features, including the antagonism of *N*-methyl-d-aspartate receptor and its impact on serotonergic neurotransmission [92,93].

Zinc serves as a cofactor for numerous enzymes and acts as a neuromodulator, influencing synaptic transmission partly through its interaction with *N*-methyl-d-aspartate and other ionotropic receptors [94]. Pharmacological studies have shown that zinc demonstrates both agonist and antagonist-like effects at the 5-HT1A receptor dependent on molar concentrations, and this dual mechanism has been proposed to underlie the antidepressant-like activity [95], acting as a negative allosteric inhibitor of 5-HT7 receptor [96]. Furthermore, inhibition of heterodimerization of 5-HT1A receptor and galanin receptor 1 can be achieved with zinc supplementation, typically at doses of 25–50 mg elemental zinc per day in human trials [97], with the involvement of G protein-coupled receptor 39, known as the zinc-binding receptor [98], all of which have been suggested as potential therapeutic targets for depression.

### Phytochemicals

Beyond traditional micronutrients, a growing body of research indicates that bioactive plant compounds, or phytochemicals, can also influence the serotonergic system. Flavonoids, a large class of polyphenols found in fruits, vegetables, tea, and wine, have demonstrated potential to modulate serotonin pathways. Certain flavonoids, such as quercetin and kaempferol, have been shown in preclinical models to exert weak MAO-A inhibitory effects, which would slow the degradation of serotonin and increase its synaptic availability [99,100]. Furthermore, many phytochemicals possess anti-inflammatory and antioxidant properties, which can indirectly support serotonin synthesis by reducing the activity of the inflammation-sensitive indoleamine 2,3-dioxygenase (IDO) enzyme and protecting the cofactor BH_4_ from oxidation [101]. Curcumin, the active compound in turmeric, has also been studied for its antidepressant effects, which may partly be attributed to its ability to increase concentrations of serotonin and other neurotransmitters in key brain regions, inhibit MAO-A, and interact with 5-HT1A/1B and 5-HT2C receptors, with the dose range tested from 10 to 80 mg/kg [[102], [103], [104]]. Although promising, evidence is largely restricted to preclinical studies, and more human clinical trials are needed to determine the efficacy and optimal dosage of these compounds for modulating the serotonergic system for health.

## The Gut Microbiota: A Modulator of the Serotonergic System

The gut microbiota, comprising the vast and complex community of microorganisms residing within the gastrointestinal tract, has emerged as a regulator of host physiology, brain function, and behavior and is known to engage in the bidirectional communication network known as the “microbiota-gut-brain axis” [105,106]. Serotonin serves as an important node at both terminals of this network, and it is becoming clear that the gut microbiota modulates the serotonergic system at multiple levels [107].

### Microbiota and peripheral serotonin

**FIGURE 3:**
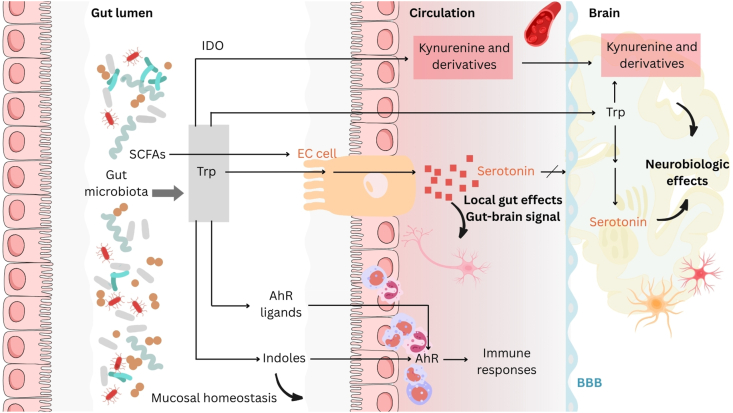
The role of the gut microbiota in the generation of l-tryptophan (Trp) catabolites, including serotonin, which can influence host physiology. The gut microbiota can modulate the availability of Trp and contribute to several metabolic pathways: the serotonin, the indole and aryl hydrocarbon receptor (AhR) binding, and kynurenine pathways. Trp can be transformed into serotonin within enterochromaffin (EC) cells. Peripheral serotonin does not cross the blood-brain barrier (BBB) but has local effects including gastrointestinal motility and can communicate with the brain via the vagus nerve. Gut microbiota-derived products such as short-chain fatty acids (SCFAs) can stimulate EC cells to produce serotonin. The serotonin pathway in the brain contributes to neurobiologic effects along with kynurenine and derivatives derived from Trp by the enzyme indoleamine 2,3-dioxygenase (IDO). Kynurenine and derivatives exist peripherally and centrally, playing a role in inflammation and immune responses. Trp can also be converted to indoles and AhR ligands acting on AhR found in intestinal immune cells to shape host immune and mucosal homeostasis.

The principal source of peripheral serotonin is EC cells, located within the gut epithelium, interfacing the host metabolic pathways and the gut microbiota. The ability to synthesize serotonin de novo may be found in certain bacteria, including *Lactobacillus plantarum* [108] and *Escherichia coli* K-12 [109], and has been suggested to be evolutionarily advantageous [110]; however, the evidence thus far has been restricted to in vitro models with unknown physiological relevance in the human gut [111]. Alternatively, the contribution of the gut microbiota to host serotonin concentrations is increasingly recognized, including certain spore-forming bacteria such as *Clostridium ramosum*, which have been found to stimulate EC cells to synthesize serotonin [6,112]. This stimulation appears to be mediated, at least in part, by microbial metabolites generated from the fermentation of dietary fiber, most notably short-chain fatty acids (SCFAs) such as butyrate, propionate, and acetate [113,114]. These SCFAs can act directly on EC cells, influencing *Tph1* gene expression and ultimately triggering serotonin synthesis and release as well as modulation of gastrointestinal motility [113]. Furthermore, the gut microbiota itself possesses extensive metabolic capabilities regarding Trp (Figure 3). Endogenous enzymes or microbial metabolism can divert Trp away from host serotonin synthesis toward alternative pathways, primarily the kynurenine pathway (leading to the production of neuroactive kynurenine metabolites) via IDO or the indole pathway [generating indole and related products or aryl hydrocarbon receptor ligands], implicated with the immune system to modulate gut homeostasis [115,116]. Beyond the substrate availability, a recent study has demonstrated that indole and its derivative, indole-3-carboxyaldehyde, produced from the Trp metabolism of *Edwardsiella tarda* can activate transient receptor potential ankyrin 1 in EC cells to enhance serotonin secretion and stimulate enteric and vagal neuronal pathways, establishing a mechanistic link in which microbial signals can be transmitted to the nervous system [117]. Together, the role of the gut microbiota in determining the host Trp pool, in tandem with its impact on EC cells, may exert a broad influence on the biosynthesis of serotonin and microbe–host communication.

### Microbiota and central serotonin

Although serotonin from the peripheral pool does not readily cross the BBB to directly influence CNS function, the gut microbiota can exert considerable impact on central serotonin systems through several distinct mechanisms [118]. First, as mentioned above, microbial Trp metabolism is a determinant of systemic Trp availability. By competing with the host for dietary Trp or by generating metabolites that influence host Trp pathways, the microbiota can alter the amount of Trp that reaches the circulation and is subsequently available for transport into the brain, thereby impacting CNS serotonin synthesis capacity [119]. Second, the gut microbiota and its metabolites can communicate with the brain via activation of afferent vagal nerve pathways. Signals originating from the gut lumen can be transmitted through the vagus nerve to the brainstem, influencing activity in central neurotransmitter systems, including the serotonergic raphe nuclei [120]. Third, the gut microbiota plays a crucial role in priming and modulating the host immune system through its impact on the production of both proinflammatory and anti-inflammatory cytokines [121]. An imbalance favoring proinflammatory cytokines can, in turn, upregulate the activity of IDO in peripheral tissues and the brain. IDO activation shunts Trp metabolism down the kynurenine pathway, effectively reducing the availability of Trp for serotonin synthesis, a mechanism implicated in the link between inflammation and depression [122,123]. Finally, microbial metabolites, such as SCFAs, can influence the integrity and permeability of the BBB. Gut dysbiosis has been associated with increased BBB permeability (“leaky barrier”), which could potentially disrupt the serotonergic system in maintaining brain homeostasis and overall functional outcomes [124]. Local gut environment and associated microbial population abundance may further fine-tune SCFA production, resulting in differences in serotonin release from mucosal cells and activation of serotonin receptors in the central compartment [125].

### Dietary modulators of the gut microbiota and serotonin

Dietary strategies aimed at modulating microbial composition and function represent a promising avenue for influencing serotonergic pathways, given that diet itself acts as a primary driver shaping the gut microbial ecosystem [126]. Specific dietary components relevant to serotonin and related pathways have been uncovered, including prebiotics, defined as nondigestible dietary fibers (e.g., inulin, fructooligosaccharides, and galactooligosaccharides) that stimulate the growth and/or activity of certain gut bacteria such as *Lactobacillus* and *Bifidobacteria* [127]. This modulation of the gut environment may offer new avenues for reducing depression-like behavior and anxiety [128,129]. However, the mechanisms by which prebiotics support behavior remain unknown but may influence serotonin production by providing SCFAs, which can stimulate *Tph1* gene expression in EC cells [113] and through the gut–brain axis, thereby exerting anxiolytic or antidepressant-like effects [128]. In addition, probiotics, which are live microorganisms administered in adequate amounts to confer a health benefit on the host, have garnered great interest in the context of health promotion [130] and may extend to its impact on serotonin regulation. Certain probiotic strains, sometimes termed “psychobiotics,” have demonstrated the ability to modulate brain neurochemistry, including serotonin concentrations, and improve behavioral outcomes related to mood and stress responses in preclinical models and some human clinical studies [131,132]. For example, specific strains such as *L. rhamnosus* (e.g., strain JB-1) have been shown to alter γ-aminobutyric acidergic signaling via the vagus nerve [120], whereas *Bifidobacterium infantis* (e.g., strain 35624) demonstrated the ability to normalize stress-induced alterations in Trp metabolism in animal models [133]. These strains may be available as targeted dietary supplements or found in fermented foods, which are defined by the International Scientific Association for Probiotics and Prebiotics as foods made through desired microbial growth and enzymatic conversions of food components [134].

Fermented foods represent whole food-based microbiota-modulating interventions culturally widespread across cuisines (e.g., kimchi/miso/natto in East Asia and yogurt/kefir/sauerkraut in Mediterranean/Eastern Europe) and can influence the serotonergic system [135]. Mechanistically, fermented foods can *1*) supply specific taxa (lactobacilli and bifidobacteria) capable of upregulating EC cell TPH1 and serotonin synthesis; *2*) increase SCFAs that stimulate serotonin release from EC cells; *3*) deliver Trp and microbially transformed indoles that engage host receptors (e.g., aryl hydrocarbon receptor) and immune pathways impacting the Trp–kynurenine balance; and *4*) enhance barrier integrity and vagal signaling within the microbiota–gut–brain axis [135]. Preclinical work with kefir demonstrates shifts in colonic serotonin turnover alongside behavioral effects [136], and an initial human “psychobiotic diet” trial enriched in prebiotic and fermented foods reported reduced perceived stress with greater benefits when adherence to the diet was higher [137]. However, these effects were only observed within the intervention group [137], and in general, human studies investigating serotonin modulation in the context of fermented foods remain limited; thus, caution is warranted in interpreting the existing data.

Furthermore, the early life period is increasingly recognized to be a critical window during which the establishment and maturation of the gut microbiota can be shaped by environmental factors, including maternal consumption of nutrients [138]. Perturbations to the gut microbiota during this highly dynamic period may be sources of disease later in life, offering mechanistic insights within the conceptual framework of early life programming as described by the Developmental Origins of Health and Disease hypothesis [139]. Emerging research highlights the vulnerability of the developing serotonergic system to various nutritional influences [[140], [141], [142]]. For example, intrauterine growth-restricted rat offspring exposed to a high-fat high-sucrose diet postnatally had higher serotonin-producing *Streptococcus*, along with greater grain serotonin availability, which may be a compensatory response to a reduction in serotonin and SERT in the maternal calorie-restricted environment [141]. In a different study, gestational exposure to a 10-fold increase in folic acid - either alone or in combination with removal of the essential nutrient choline - resulted in lower 5-HT2C receptor protein expression in the hypothalamus and disruptions in refeeding response to a 5-HT2C receptor agonist [142]. Brain serotonin concentrations were lower, whereas colon serotonin concentrations were higher, consistent with obesogenic phenotypes [140,142], with specific taxa, including *Lactococcus*, *Ruminococcus*, *Bacteroides*, and *Oscillospira*, that distinguished the effects of the dietary groups [142]. Together, time- and/or sex-specific alterations in gut microbiota composition have been reported, with potential implications for divergent effects of diet on the serotonergic system that may confer differential risk in long-term neurological and behavioral outcomes [141,142].

## Clinical Implications of Nutritional Modifiers of Serotonin

The nutritional modulation of serotonin synthesis and function carries considerable implications for a wide range of clinical conditions, in which the serotonergic system may be a viable target for therapeutics. Emerging evidence has established a link between dysfunctions in serotonin and mood disorders including depression and anxiety [143], sleep disorders [144], disordered eating and obesity [145], and irritable bowel syndrome [146] (Table 1).

**TABLE 1:** Summary of potential nutritional impact on serotonin-related outcomes.

Intervention	Target mechanism	Potential clinical application(s)	Example dosing/duration/study population	Key findings
Trp/5-HTP supplementation	Increase precursor availability	Mood and sleep disorders	Trp: 0.5–6 g/d; 5-HTP: 50–300 mg/d. Duration: 2–8 wk. Population: adults with mild–moderate depression or insomnia (*n* = 20–100).	Mixed results for depression; some evidence for sleep.
High carbohydrate	Increase Trp/LNAA ratio, brain Trp uptake/synthesis	Disordered eating and obesity; mood and sleep disorders	Acute meal studies. Population: healthy adults or individuals with mood/sleep complaints (*n* = 10–50).	May contribute to satiety; some studies show mood/sleep benefits.
ω-3 PUFA supplementation (EPA/DHA)	Modulate membrane fluidity, receptor modulation, and lipid mediators	Mood disorders	EPA-rich formulas (>60% EPA) at ∼1–2 g/d. Duration: ≥8 wk. Population: adults with MDD (*n* = 40–200).	Meta-analyses suggest benefit for MDD, particularly EPA.
B-vitamin supplementation (vitamins B6, B9, B12)	Restore cofactor availability for serotonin synthesis; activation of serotonergic neurons	Mood disorders	B6: 50–100 mg; folate (vitamin B9): 400–800 μg; vitamin B12: 500–1000 μg. Duration: weeks to months. Population: individuals with deficiencies or elevated homocysteine.	Supplementation may benefit specific populations.
Vitamin D supplementation	Potentially regulate TPH1 and TPH2 expression	Mood disorders	2000–5000 IU/d. Duration: >8 wk. Population: individuals with vitamin D insufficiency (<30 ng/mL) and depression.	Supplementation may benefit specific populations.
Iron/magnesium/zinc supplementation	Restore cofactor availability for serotonin synthesis; modulator of serotonin receptor or neurotransmission	Mood disorders	Dose depends on deficiency level (e.g., Mg: 200–500 mg/d). Duration: ≥4 wk. Population: individuals with diagnosed deficiency.	Supplementation may benefit specific populations.
Prebiotics/dietary fiber components	Modulate gut microbiota, increase SCFAs (via gut–brain axis)	IBS, mood disorders	5–15 g/d of fibers such as inulin, fructooligosaccharides, and galactooligosaccharides. Duration: ≥4 wk. Population: healthy adults, patients with IBS or anxiety (*n* = 30–100).	Improvement in gut health; emerging evidence for antidepressant-like effects.
Probiotics (“psychobiotics”)	Modulate gut microbiota, gut–brain axis signaling	IBS, mood disorders	Strain-specific, 10^9^–10^10^ CFU/d. Duration: ≥4 wk. Population: adults with IBS, anxiety, or depression (*n* = 40–150).	Strain-specific effects; promising results for gut health and improvement in mood and stress responses.
Mediterranean diet	Multiple (Trp sources, micronutrients, fiber, PUFAs)	Mood disorders	Adherence to dietary pattern. Duration: ≥12 wk (intervention trials). Population: adults with moderate-to-severe depression (e.g., SMILES trial, *n* = 67).	Inverse association with depression; intervention trial results supportive.
Imbalances in macronutrients or micronutrients in early life period	Modulate gut microbiota; gut–brain axis	Disordered eating and obesity; mood disorders	Observational and preclinical studies of maternal diet.	Long-term programming of neurological and behavioral outcomes toward higher disease risk

Abbreviations: 5-HTP, 5-hydroxytryptophan; CFU, colony-forming units; DHA: docosahexaenoic acid; EPA: eicosapentaenoic acid; IBS, irritable bowel syndrome; LNAA, large neutral amino acids; MDD, major depressive disorder; PUFA: polyunsaturated fatty acid; SCFA, short-chain fatty acid; SMILES, Supporting Modification of Lifestyle in Lowered Emotional States; TPH, tryptophan hydroxylase; Trp: L-tryptophan.

### Mood disorders (depression and anxiety)

The “monoamine hypothesis” of depression, initially proposed decades ago, posited that a functional deficiency in brain serotonin (along with norepinephrine and dopamine) contributes significantly to the etiology of depressive symptoms [147]. Although now recognized as an oversimplification of the complex pathophysiology of depression, the serotonin system remains a key therapeutic target for many widely prescribed antidepressant medications, primarily SSRIs. Evidence supporting the role of serotonin in brain function arises from acute Trp depletion (ATD) studies [148]. This experimental paradigm, typically using a double-blind, placebo-controlled crossover design, involves administering a nutritional drink containing a balanced mixture of essential amino acids but lacking Trp to participants after an overnight fast. This floods the LAT1 transporter at the BBB with competing LNAAs, thereby transiently lowering brain Trp influx and serotonin synthesis by ≤90% [149]. In a study with 20 participants (45% female) with remitted depression and who had previously responded well to SSRI treatment, administration of a 100-g amino acid mixture designed to reduce Trp concentrations by 90% induced a temporary relapse of depressive symptoms, whereas these effects were largely absent with a 50-g dose [150]. Sex effects were also observed, with female participants exhibiting a more pronounced response than males [150]. A meta-analysis across 45 studies further showed that ATD can lead to mood deterioration in healthy individuals with a positive family history and in patients with remitted depression, with the latter group showing more prominent effects [151]. In contrast, healthy individuals without predispositions for depression did not show mood changes [151], suggesting that ATD may selectively reveal serotonergic vulnerability in at-risk groups. Similarly, a more recent meta-analysis found only weak mood effects in individuals with a family history of depression (*n* = 75) and no consistent evidence in healthy individuals (*n* = 566) [152]. However, the role of ATD in anxiety disorders remains inconclusive, as highlighted by a systematic review that noted heterogeneity across studies, many of which were limited by small sample sizes [153].

Supplementation with the direct precursors, 5-HTP or Trp, has also been raised as a potential strategy for modulating brain function [154]. Notably, 5-HTP bypasses the rate-limiting TPH step and readily crosses the BBB, potentially offering a more direct route to increasing serotonin synthesis, with efficacy comparable with conventional antidepressants in some studies [155]. A recent randomized, double-blinded, placebo-controlled crossover study with 77 healthy adults (51% female) challenged with an acute 200-mg 5-HTP intake followed by employing a mixed between-subject design to provide a diet containing 500-mg Trp/d for 4 wk showed that 5-HTP or Trp may positively influence the recognition of positive emotions and better integration of information [156]. However, results remain mixed, and historical concerns regarding potential contaminants (leading to eosinophilia-myalgia syndrome with Trp supplements in the past) necessitate caution [154,157], in addition to BBB breakdown, edema, and neuronal disturbances associated with 5-HTP-induced elevation of plasma and brain serotonin concentration [158]. The variability in study outcomes is likely due to heterogeneity in patient populations, small sample sizes, differences in dosage and study duration, and a frequent lack of control for the availability of essential micronutrient cofactors required for the conversion of these precursors to serotonin.

As detailed previously, B-vitamins (B6, B9, and B12), vitamin D, iron, magnesium, and zinc are of interest as key micronutrients involved in serotonin metabolism; thus, deficiencies in these micronutrients are plausible contributors to the pathophysiology of depression [159,160]. Correcting these deficiencies through targeted supplementation, particularly in individuals with intake concentrations below the recommendation or low micronutrient status, may improve mood, often when used as an adjunct to standard antidepressant therapies [[161], [162], [163]]. Supporting this concept, micronutrient supplementation, for example, magnesium (at doses of 200–500 mg/d), alone or together with another micronutrient vitamin B6 (50–100 mg/d), has been suggested to confer clinical benefits for the symptomatic treatment of mood and anxiety, as measured by improvements on scales using the Depression Anxiety Stress Scales and Short Form-36 Health Survey [164]. Although these questionnaires are validated for clinical conditions and quality of life, their self-reported nature, combined with the absence of a placebo group, necessitates careful consideration when interpreting the results. ω-3 PUFA supplementation, especially formulations rich in EPA, has also shown promise, with meta-analyses suggesting potential antidepressant effects, particularly in individuals diagnosed with MDD [165,166]. A recent preliminary study also reported that DHA and ω-3 status were negatively associated with depressive and anxiety rating scores in young adults with subthreshold depression, attributed to reduced connectivity between the orbitofrontal cortex and angular gyrus [167], whereby greater connectivity patterns have been found with lower serotonin concentration in patients with MDD compared with healthy controls [168]. Dietary pattern is also of interest, as epidemiological studies have shown an inverse association with adherence to the Mediterranean diet and risk of developing depression [169]. The landmark Supporting Modification of Lifestyle In Lowered Emotional States trial has also demonstrated that targeted dietary improvement programs involving whole foods, such as those emphasized in a modified Mediterranean diet (e.g., vegetables, fruits, whole grains, legumes, fish, lean red meats, olive oil, and nuts), can significantly reduce depressive symptoms in individuals with moderate-to-severe depression [170], emphasizing the overall diet quality in mood management. The mechanisms by which the Mediterranean diet may confer these benefits are multifactorial but likely involve modulation of the serotonergic system. The diet has been shown to increase the abundance of fiber-fermenting bacteria, including *Lachnospiraceae* and *Butyricicoccus*, with potential protection of the Trp pool from depletion via the IDO pathway [171]. Additionally, the high fiber content of the Mediterranean diet is thought to exert prebiotic effects by promoting the growth of certain microbes such as *Lactobacillus* [172], potentially contributing to enhanced serotonin biosynthesis [173].

### Sleep disorders

Serotonin serves as the direct biochemical precursor for melatonin, the principal hormone responsible for regulating circadian rhythms and sleep–wake cycles, which is synthesized primarily in the pineal gland [174]. Consequently, nutritional factors influencing serotonin availability (including dietary Trp) or synthesis can potentially impact melatonin production and sleep quality. Supplementation with Trp has been shown in some studies to improve subjective measures (e.g., self-reported ease of falling asleep, restedness upon waking, and overall sleep satisfaction) and objective measures of sleep quality, such as reducing sleep latency (time taken to fall asleep objectively measured by polysomnography or actigraphy) and increasing total sleep duration, particularly in individuals experiencing mild insomnia or poor sleep [175,176]. Certain foods (e.g., milk, turkey, and chicken) that are naturally rich in Trp, α-lactalbumin in whey as a stimulator of Trp/LNAA ratio, kiwifruit, which is known to contain serotonin, and tart cherries, which are a dietary source of Trp, serotonin, and melatonin, have been of interest for improving sleep outcomes [177]. However, the fact that the quantity of melatonin in these dietary sources is in the microgram range, as opposed to the milligram doses typically used to treat insomnia, suggests that their beneficial effects on sleep quality likely extend beyond the direct action of ingested melatonin. Instead, these foods may support the body’s endogenous synthesis of serotonin and melatonin via their content of Trp and other synergistic nutrients [178]. Trp degradation serves as another dimension, as the metabolic product of Trp breakdown mediated by IDO is kynurenine, with the kynurenine/Trp ratio being the gold standard for determining IDO activity. A recent pilot study has proposed that the procyanidin B-2 in tart cherry juice reduced the ratio of kynurenine/Trp, reflecting its action as an inhibitor of IDO, which may improve sleep efficiency through its impact on sustaining Trp bioavailability for serotonin synthesis [179]. Additionally, the timing and composition of meals may play a role, as chrononutrition is an emerging field of study that investigates the relation between eating patterns and circadian rhythms [180]. Consuming high-glycemic-index carbohydrate meals a few hours before bedtime has been suggested to improve sleep latency, potentially by leveraging the insulin-mediated increase in the brain Trp/LNAA ratio, thereby enhancing serotonin and subsequent melatonin synthesis [181]. Thus, serotonin and related pathways are gaining recognition as having important roles in the relation between dietary patterns and sleep but will require further investigations going beyond small-scale short-term studies to confirm these findings.

### Disordered eating and obesity

The expansive nature of the serotonergic system encompasses its role in appetite control and energy balance and has garnered interest in serotonin as a potential therapeutic target to modulate feeding behavior. Serotonin is primarily considered an anorexigenic (appetite-suppressing) signal that involves key hypothalamic nuclei such as the arcuate nucleus, paraventricular nucleus, and ventromedial hypothalamus [182], with integration of metabolic inputs supporting the homeostatic regulation of energy needs. The previously discussed mechanism by which carbohydrate intake increases brain serotonin synthesis may contribute physiologically to feelings of satiety and fullness after a meal [49]. However, the interplay between carbohydrates, serotonin, and appetite remains complex, wherein chronic dysregulation of the serotonergic system has been hypothesized to contribute to phenomena such as “carbohydrate-craving” and difficulties with weight management in some individuals [183]. Rodent models of hyperenergetic diet-induced obesity have shown region-specific alterations in the binding capacity of 5-HT1A, 5-HT1B, and 5-HT2A receptors in the hypothalamus, consistent with reduced serotonin release and lower activity of the serotonergic neurons [184], as well as overall abolishment of serotonergic mechanisms [185], suggesting perturbations in serotonin in relation to the propensity to obesity. Pharmacological agents designed to augment the appetite-suppressing actions of serotonin (e.g., d-fenfluramine, which increases the release of serotonin, or the 5-HT2C receptor agonist lorcaserin) have historically been developed and utilized as antiobesity treatments [186,187], although some were later withdrawn because of safety concerns related to off-target effects [188]. Attention is growing with regard to the role of serotonergic signaling in the hedonic reward neurocircuitry that interacts with dopaminergic signaling to impact motivational food consumption and the effect of peripheral serotonin on stimulation of energy absorption and storage [145], highlighting multiple networks beyond the hypothalamic regulatory pathways. New strategies that target serotonin receptor subtypes thus will require consideration of the physiology of various subregions of the brain and organ systems to improve the efficacy and peripheral side effect profiles.

### Irritable bowel syndrome

Given that the vast majority of the body’s serotonin is produced and has local effects within the gastrointestinal tract, where it regulates motility, secretion, and visceral sensation (pain perception), it is unsurprising that dysregulation of gut serotonin has been of special interest in the pathophysiology of IBS [189]. Numerous studies have reported alterations in various aspects of serotonin signaling in patients with IBS compared with healthy controls, including changes in Trp metabolism pathways, altered numbers or function of EC cells, differences in mucosal or systemic serotonin concentrations, and variations in the expression or function of SERT, which is responsible for serotonin reuptake [190]. From a nutritional standpoint, the management of IBS often involves dietary modification including diets low in fermentable oligo-, di-, monosaccharides, and polyols (FODMAPs), which have been shown to alleviate symptoms such as abdominal pain, bloating, and altered bowel habits [191,192]. These benefits are thought to arise partly from reducing osmotic load and gas production due to fermentation [192] but may also involve modulation of microbial activity and SCFA profiles, which could secondarily impact gut serotonin signaling [193]. A recent report revealed the EC cell-mucosal afferent circuit to be an important link to visceral hypersensitivity and necessary for the sensitizing actions of isovalerate, a SCFA associated with gastrointestinal inflammation, with potentially greater tonic input present in females than in males [194]. Additional complexity occurs with the variability in metabolic traits among individuals with IBS [195,196], which may influence responsiveness to a low FODMAP diet. Responders have been shown to display unique fecal SCFA profiles that correlated with elevated urinary Trp and 5-HIAA concentrations, suggesting that modulation of the SCFA-serotonin axis may underlie symptom relief [195]. Furthermore, FODMAP restriction appears to reduce SCFAs in the “pathological” metabotype but not in the “healthy” metabotype, correlating with bacterial metabolic pathways [196]. Specific probiotic strains, such as *B. infantis* 35624 or *L. plantarum* 299v, have been shown to improve global IBS symptoms, including bloating and abdominal pain [197,198], supporting the emerging concept of the serotonin–probiotics–gut health axis [199]. Overall, the broader system of gut serotonin signaling and associated pathways makes discerning molecular and functional mechanisms in gastrointestinal disorders important, as they may provide promising directions for the development of effective therapeutic strategies.

## Challenges and Gaps in Knowledge

Despite considerable progress in elucidating the relation between nutrition and serotonin, significant challenges and knowledge gaps remain, hindering the straightforward translation of current findings into clinical applications. The inherent complexity of diet, comprising a mixture of nutrients and nonnutrient bioactive compounds, makes isolating the specific effects of individual dietary components on serotonin pathways within the context of a whole diet exceptionally difficult. Nutrient–nutrient interactions are inevitable; for example, B-vitamins often work synergistically in metabolic pathways relevant to serotonin [200]. Furthermore, substantial individual variability exists in response to nutritional interventions targeting serotonin [201]. Genetic factors, such as polymorphisms in genes encoding key enzymes (e.g., TPH1 and TPH2), SERTs, or metabolic enzymes (e.g., MAO-A), can influence serotonin function and responsiveness to dietary changes [202]. Baseline nutritional status, gut microbiota composition, age, sex, stress levels, and underlying health conditions further contribute to this variability, strongly suggesting that personalized nutrition approaches will likely be necessary for optimal efficacy.

Moreover, the vast majority of research has been conducted in North American and European populations. Dietary patterns, genetic polymorphisms in serotonin-related genes, and gut microbiota compositions can vary across different geographical and ethnic groups. Despite distinct culinary staples, both traditional Asian (e.g., kimchi, miso, and natto) and Mediterranean/Eastern European (e.g., yogurt, kefir, and sauerkraut) diets incorporate fermented foods that deliver live microbes and metabolites with plausible effects on serotonin. Future multicultural trials could quantify fermentation exposure (live microbe dose, product category, microbial composition, and postbiotic content) and test serotonergic readouts (e.g., Trp/LNAA ratio, fecal/urinary 5-HIAA, gut microbiota–brain axis-linked metabolomics, and mood outcomes). Importantly, future studies must incorporate more diverse populations to determine the universality of these findings and to explore how region-specific diets, such as traditional Asian compared with Mediterranean diets, differentially impact the serotonergic system.

Methodological challenges also persist. Direct and dynamic measurements of serotonin synthesis rates or neurotransmitter concentrations within the human brain remain invasive, remain technically demanding, or cannot be achieved within the boundaries of study ethics. Consequently, researchers often rely on peripheral biomarkers (e.g., plasma Trp/LNAA ratio, urinary 5-HIAA excretion, and platelet serotonin content) as proxies for central serotonin status, but the degree to which these peripheral measures accurately reflect CNS serotonergic activity is often uncertain and context-dependent. Advanced neuroimaging techniques, such as positron emission tomography using specific radioligands targeting serotonin receptors or transporters, offer valuable noninvasive insights but are expensive and not widely accessible for large-scale studies. Additionally, translating findings from preclinical animal models, in which many mechanistic insights are generated, to human physiology and clinical outcomes requires careful consideration and validation. The long-term effects of sustained dietary changes on serotonin homeostasis and the potential for adaptive or compensatory mechanisms to occur over time are also not fully understood. Finally, although the crucial role of the gut microbiota is increasingly recognized, much work remains to identify the specific bacterial species, their relevant metabolites, and the precise molecular mechanisms through which they influence host serotonin regulation in both the gut and the brain. Disentangling the relative impact of diet on microbial Trp metabolism compared with direct host Trp utilization will require further studies that manipulate the gut microbiota composition.

**FIGURE 4:**
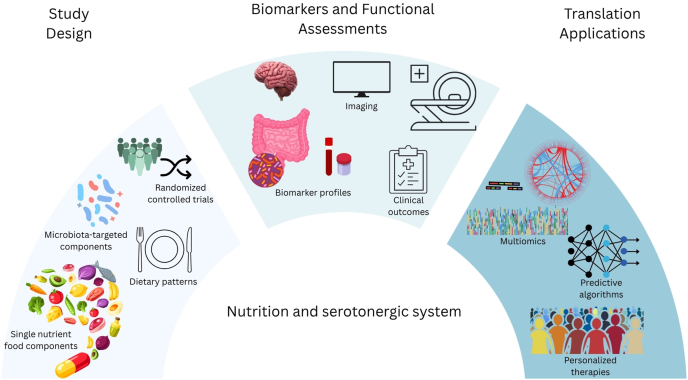
Potential approaches to harness the serotonergic system using nutrition. Study design: nutritional and/or microbiota-targeted interventions (including single nutrient/food components, and whole dietary patterns), prebiotics, probiotics, synbiotics, or postbiotics should be implemented in well-powered randomized controlled trials to determine the extent to which the host serotonin can be modulated for therapeutic benefits. Biomarkers and functional assessments: robust, validated outcome measures of serotonin metabolism and clinical outcomes such as mood, sleep, gut function, and physical health should be incorporated in studies that focus on the effect of singular nutrients, with an expansion to define the complex, potentially synergistic effects of whole dietary patterns, on serotonin homeostasis and well-being. Neuroimaging techniques, including positron emission tomography, may provide direct assessment of central serotonergic changes. Translational applications: multimomics approaches encompassing genomics, transcriptomics, proteomics, metabolomics, and gut metagenomics can provide systems-level integration of how nutritional inputs influence serotonin pathways and related health outcomes shaped by interindividuality in biological makeup. Multidimensional readouts will be valuable in building predictive algorithms to identify specific groups of individuals most likely to benefit from targeted nutritional strategies, supporting personalized therapies for serotonin-related conditions.

Addressing these challenges necessitates focused future research efforts (Figure 4) that can pave the way for novel therapeutic applications. First, there is a critical need for more well-designed human intervention studies, specifically randomized controlled trials with adequate statistical power and clearly defined and controlled dietary interventions (ranging from single nutrient/food components to whole dietary patterns). Microbiota-targeted interventions, including specific prebiotics, probiotics, synbiotics (combinations of pre- and probiotics), or even postbiotics (beneficial microbial metabolites), will also be essential to determine their capacity to reliably modulate the host serotonergic system for therapeutic benefit. Second, studies that focus on singular nutrients should be expanded to define the complex, potentially synergistic effects of whole dietary patterns on serotonin homeostasis and overall mental and physical health, paired with the use of robust, validated outcome measures encompassing both biomarkers of serotonin metabolism and standardized clinical assessments of mood, cognition, sleep, and gut function. Wider application of advanced neuroimaging techniques such as positron emission tomography in nutritional intervention studies could provide invaluable direct evidence of central serotonergic changes. Finally, integrating multiomics approaches, combining genomics, transcriptomics, proteomics, metabolomics, and gut metagenomics, can provide a systems-level understanding of how nutritional inputs interact with an individual’s unique biological makeup to influence serotonin pathways and related health outcomes. Efforts should be directed toward developing predictive algorithms to identify specific groups of individuals most likely to benefit from targeted nutritional strategies, paving the way for personalized interventions for serotonin-related conditions.

## Future Outlook

Beyond the current research gaps, the future of nutritional impact on serotonin holds several exciting prospects. Harnessing this knowledge will move beyond generalized advice toward highly personalized and technologically integrated strategies.

Future interventions will likely involve genotyping for polymorphisms, for example, TPH2, SERT, or MAO-A, to create personalized dietary recommendations [203]. An individual with a less efficient *SERT* variant, for example, might receive targeted advice emphasizing nutrients that support serotonin synthesis (e.g., B-vitamins, and magnesium) and foods rich in ω-3 PUFAs to optimize neuronal membrane function.

The development of next-generation probiotics, or “psychobiotics,” will move beyond general gut health. Specific bacterial consortia engineered to produce serotonin precursors, modulate host IDO activity, or generate neuroactive SCFAs could be prescribed as medical foods for mood or anxiety disorders. Moreover, the direct administration of purified postbiotics (e.g., specific SCFAs or indole derivatives) may offer another strategy to achieve desired effects on the serotonergic system [204].

The development of noninvasive or minimally invasive biosensors capable of monitoring peripheral serotonin metabolites or even central neurotransmitter dynamics in real time could vastly advance nutritional interventions. Such technology would allow for a direct feedback loop, enabling individuals to see the immediate biochemical impact of a meal on their serotonergic system and adjust their diet accordingly [205,206]. Concerted efforts can also be made to move beyond essential nutrients to include bioactive phytochemicals, for which future research may systematically screen plant-derived compounds, such as specific flavonoids, for their ability to act as natural MAO inhibitors, SERT modulators, or TPH cofactors, paving the way for novel, evidence-based functional foods and nutraceuticals [101,207].

In conclusion, this review has synthesized the substantial body of evidence demonstrating the nutritional impact on serotonin and highlighted its significant clinical relevance for conditions intrinsically linked to serotonergic function. Several key points emerged: *1*) serotonin availability is nutritionally constrained by Trp intake and transport competition at the BBB (Trp/LNAA ratio), together with cofactor- (iron; BH_4_ supported by folate/B12; and vitamin B6 for decarboxylation) and riboflavin-dependent MAO-A activity; *2*) carbohydrate-driven insulin responses can transiently increase brain Trp concentration, whereas typical mixed-protein meals lower the Trp/LNAA ratio, and ω-3 fatty acids improve membrane-dependent receptor/transporter function, plausibly linking macronutrient composition with serotonin-related outcomes in depression, sleep dysregulation, obesity, and IBS; *3*) adequacy of vitamin B6, folate/vitamin B12, iron, vitamin D, magnesium, and zinc, alongside polyphenol-rich, Mediterranean dietary patterns that can influence MAO-A activity or downstream pathways, supports serotonin synthesis and signaling, whereas insufficiency may be associated with higher risk of depressive/anxiety symptoms, poor sleep, and gut dysfunction; and *4*) prebiotic fibers, probiotics, and fermented foods increase SCFAs with alterations in Trp metabolism toward EC TPH1-dependent serotonin production, linking nutrition-driven gut microbiota shifts to symptoms across IBS, mood disorders, and metabolic disease along the microbiota–gut–brain axis. Although translating these complexities into precise dietary guidelines remains challenging, nutrition holds immense promise for supporting health through its influence on the serotonergic system. Harnessing this potential would require acknowledging that, at a fundamental biochemical level, the adage “you are what you eat” resonates profoundly when considering the intricate biology of serotonin.

## Author contributions

The authors’ responsibilities were as follows – JT, LK, AS: performed the literature review, created the summary table and figures, and drafted the manuscript; CEC: conceived the main idea, critically revised the manuscript, and had final responsibility for the manuscript; and all authors: read and approved the final manuscript.

## Funding

LK was supported by a Summer Undergraduate Research Assistantship. CEC holds a Canadian Institutes of Health Research Canada Research Chair (Tier II) and has work supported by the Natural Sciences and Engineering Research Council of Canada Discovery Grant.

## Conflict of interest

The authors report no conflicts of interest.
